# Preoperative CT-based radiomics combined with tumour spread through air spaces can accurately predict early recurrence of stage I lung adenocarcinoma: a multicentre retrospective cohort study

**DOI:** 10.1186/s40644-023-00605-3

**Published:** 2023-09-07

**Authors:** Yuhang Wang, Yun Ding, Xin Liu, Xin Li, Xiaoteng Jia, Jiuzhen Li, Han Zhang, Zhenchun Song, Meilin Xu, Jie Ren, Daqiang Sun

**Affiliations:** 1 Graduate School, Tianjin Medical University, Tianjin, China; 2 Clinical School of Thoracic, Tianjin Medical University, Tianjin, China; 3 Department of Thoracic Surgery, Tianjin Chest Hospital of Tianjin University, No. 261, Taierzhuang South Road, Jinnan District, Tianjin, 300222 China; 4 Department of Imaging, Tianjin Chest Hospital of Tianjin University, Tianjin, China; 5 Department of Pathology, Tianjin Chest Hospital of Tianjin University, Tianjin, China; 6 Department of Thoracic Surgery, Tianjin Jinnan Hospital, Tianjin, China

**Keywords:** Radiomics, Lung adenocarcinoma, Preoperative CT, Deep learning, STAS

## Abstract

**Objective:**

To develop and validate a prediction model for early recurrence of stage I lung adenocarcinoma (LUAD) that combines radiomics features based on preoperative CT with tumour spread through air spaces (STAS).

**Materials and methods:**

The most recent preoperative thin-section chest CT scans and postoperative pathological haematoxylin and eosin-stained sections were retrospectively collected from patients with a postoperative pathological diagnosis of stage I LUAD. Regions of interest were manually segmented, and radiomics features were extracted from the tumour and peritumoral regions extended by 3 voxel units, 6 voxel units, and 12 voxel units, and 2D and 3D deep learning image features were extracted by convolutional neural networks. Then, the RAdiomics Integrated with STAS model (RAISm) was constructed. The performance of RAISm was then evaluated in a development cohort and validation cohort.

**Results:**

A total of 226 patients from two medical centres from January 2015 to December 2018 were retrospectively included as the development cohort for the model and were randomly split into a training set (72.6%, *n* = 164) and a test set (27.4%, *n* = 62). From June 2019 to December 2019, 51 patients were included in the validation cohort. RAISm had excellent discrimination in predicting the early recurrence of stage I LUAD in the training cohort (AUC = 0.847, 95% CI 0.762–0.932) and validation cohort (AUC = 0.817, 95% CI 0.625–1.000). RAISm outperformed single modality signatures and other combinations of signatures in terms of discrimination and clinical net benefits.

**Conclusion:**

We pioneered combining preoperative CT-based radiomics with STAS to predict stage I LUAD recurrence postoperatively and confirmed the superior effect of the model in validation cohorts, showing its potential to assist in postoperative treatment strategies.

**Supplementary Information:**

The online version contains supplementary material available at 10.1186/s40644-023-00605-3.

## Introduction

Lung cancer is still the leading cause of cancer-related death [1]. As the major pathological subtype of lung cancer, lung adenocarcinoma (LUAD) has been continuously researched. With the development of medical imaging, an increasing number of early-stage LUAD cases are being detected and treated. Currently, complete surgical resection is still the primary treatment for stage I LUAD [2]. However, studies have found that even after complete surgical resection, stage I lung adenocarcinoma still has a recurrence rate of 20–50% [3]. Early identification of patients at high risk for lung adenocarcinoma recurrence and timely adjustment of their treatment strategies are the keys to reducing the recurrence rate of early-stage lung adenocarcinoma and improving patient prognosis.

Chest computed tomography (CT) is currently the most commonly used ancillary test for the diagnosis and evaluation of lung cancer. With the rapid development of machine learning and artificial intelligence in the medical field, researchers are beginning to obtain quantitative data from medical images to assist in the diagnosis and prognosis prediction of medical diseases. There has been evidence that both tumour radiomics and peritumour radiomics can predict the diagnosis, outcome, and pathological subtype of lung adenocarcinoma [4–7].

In the new WHO classification of lung cancer published in 2015, the concept of tumour spread through air spaces (STAS) was formally introduced and defined as the spread of micropapillary clusters, solid nests, and/or single cancer cells in the alveolar cavity beyond the main tumour margin [8]. Several subsequent studies have shown that STAS is an important risk factor for recurrence after stage I LUAD resection [9, 10].

Currently, several studies point to the high accuracy of radiomics combined with histopathology for predicting clinical outcomes in oncology patients [11, 12]. However, no study has used radiomics combined with the presence of STAS to predict the recurrence of early-stage lung adenocarcinoma until now.

In this multicentre retrospective cohort study, we pioneered the combination of radiomics features from preoperative CT with the presence of STAS determined by postoperative pathology to develop and validate a RAdiomics Integrated with STAS model (RAISm) to help clinicians identify high-risk stage I LUAD patients early and adjust treatment strategies in a timely manner.

## Materials and methods

### Study design and population

**Fig. 1:**
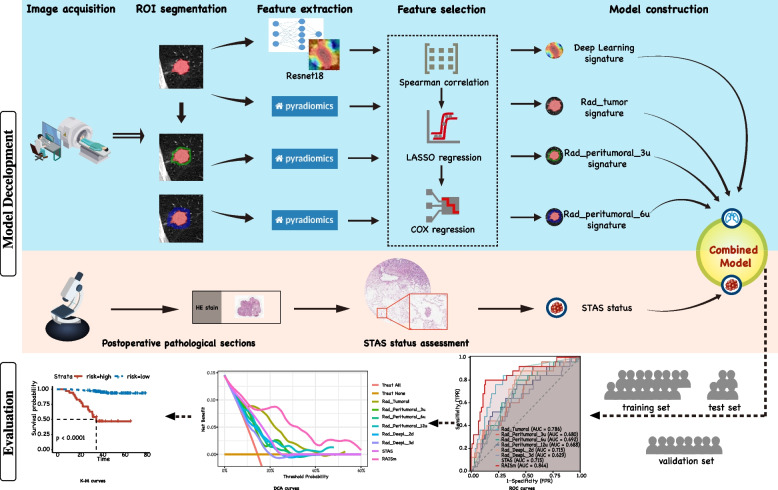
Workflow of the study. Preoperative chest CT images of patients were retrospectively collected and pre-processed, and then segmented for features extraction. Six radiomic signatures were constructed after feature selection. Postoperative haematoxylin and eosin-stained sections of patients were reviewed and assessed for STAS status and combined with radiomic signatures to construct RAISm. RAISm performance was evaluated in the training set, test set and validation set, respectively

This study consisted of a retrospective study for model development and retrospective validation in an external cohort. The study was divided into four main steps: image acquisition and processing, feature extraction and screening, STAS assessment, and model construction and evaluation (Fig. 1).

In this study, we reviewed information on patients who received treatment at Tianjin Chest Hospital and Tianjin Jinnan Hospital between 1 January 2015 and 31 December 2018 and included patients who met the following inclusion criteria: (i) underwent complete surgical resection of a lung lesion at Tianjin Chest Hospital or Tianjin Jinnan Hospital; and (ii) had postoperative pathology confirming invasive stage I LUAD. The exclusion criteria were as follows: (i) multiple primary cancers in the lung; (ii) preoperative neoadjuvant therapy; (iii) lost to follow-up after surgery; (iv) unable evaluate the presence of STAS according to postoperative pathological slices; and (v) missing, inaccessible or lack of preoperative thin-layer CT files. After screening, 226 eligible patients were eventually included in the study. After a 7:3 ratio random split, 162 patients were included in the training cohort for model construction, and 64 patients were included in the test cohort for internal validation. The purpose of internal validation as part of model development is to check the repeatability of the model development process and to prevent overfitting of the model leading to overestimation of the model performance.

**Fig. 2:**
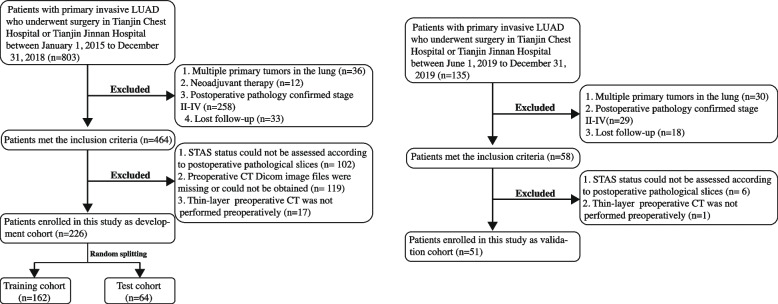
Patient selection process

The information of patients who received treatment in Tianjin Chest Hospital or Tianjin Jinnan Hospital between 1 June 2019 and 31 December 2019 was screened using the same inclusion and exclusion criteria, with 51 eligible patients eventually included in the study as the external validation cohort. Figure 2 shows the inclusion and exclusion criteria and process in detail.

### Image acquisition and preprocessing

The most recent preoperative chest CT scans in Digital Imaging and Communications in Medicine (DICOM) format were downloaded from the Picture Archiving and Communication System (PACS) of Tianjin Chest Hospital and Tianjin Jinnan Hospital.

The CT images were preprocessed because various CT scanners were used in the hospital (including PNMS, Siemens and Philips), and there were differences in layer thickness, voxel size, window width and window level among patients. We first resampled all the CT images and standardized the voxel units to 0.7*0.7*1.5. Afterwards, we standardized the window width and window level to 1350 and -350, respectively, which we found to be appropriate in the segmentation process of the region of interest (ROI).

### Region of interest segmentation and feature extraction

Two experienced thoracic surgeons (L.X, with 13 years of experience in thoracic oncology, and D.Y, with 5 years of experience in thoracic oncology) and one experienced radiologist (S.Z.C, with 15 years of experience in medical imaging) performed the fully manual segmentation of the ROI (ROI-tumoral). The tumour contours were outlined in each of the three orthogonal planes and integrated by the software into a three-dimensional structure. Any disagreements during the segmentation process were confirmed and guided by radiologist S.Z.C, and thoracic surgeon S.D. (with 30 years of experience in cardiothoracic surgery) reviewed the segmentation results. ROI segmentation was performed using the open-source software ITK-SNAP (version 3.8.0).

**Fig. 3:**
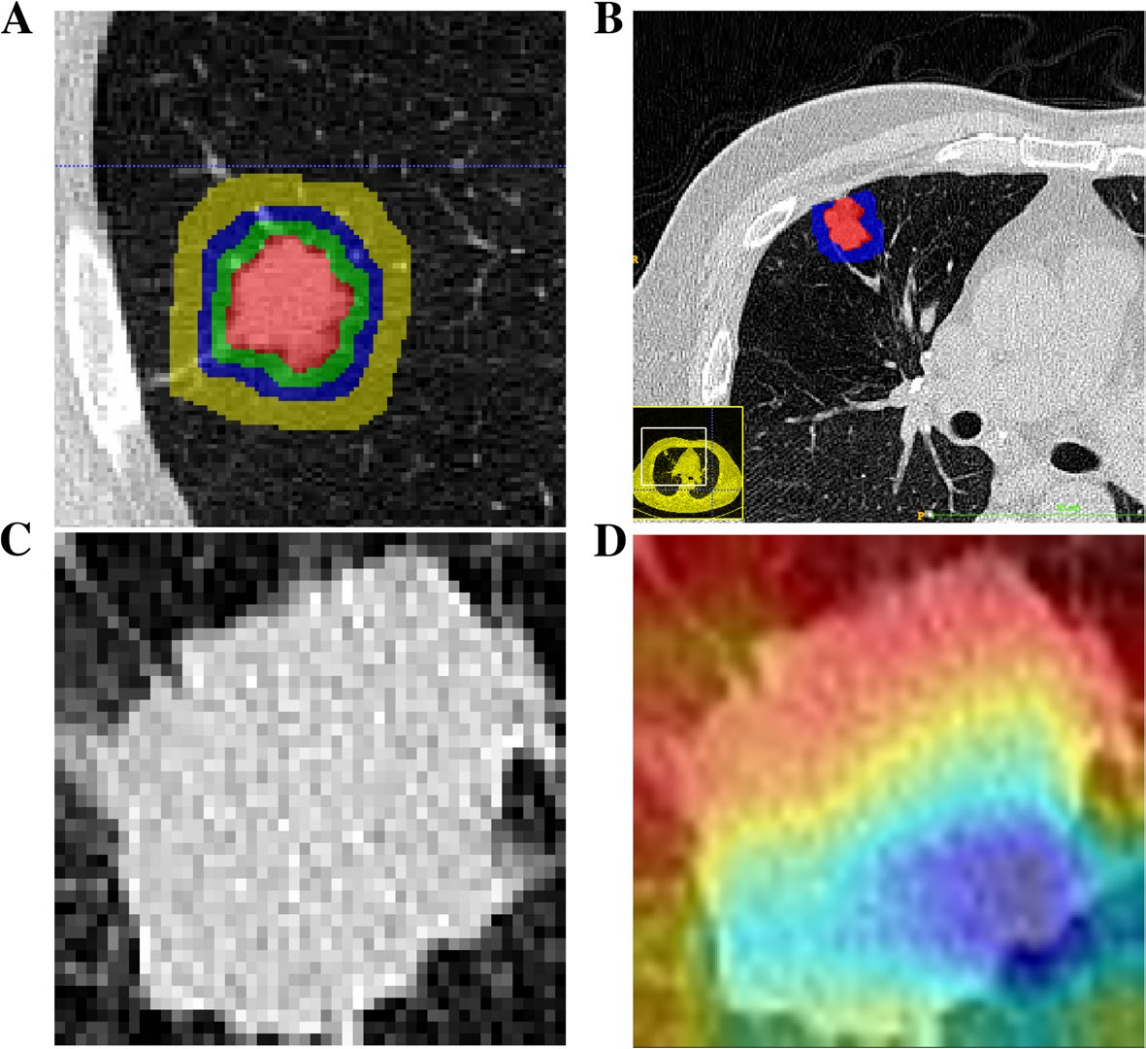
Region of interest segmentation and feature extraction. **A** The tumor on the CT image was manually segmented and ROI-tumoral was constructed. 3 voxel units, 6 voxel units and 12 voxel units were respectively amplified outward to construct ROI-3u, ROI-6u and ROI-12u based on the ROI-tumoral. **B** Peritumoral areas that expand beyond the lung parenchyma are erased to prevent errors. **C**-**D** The maximum cross-section of ROI-tumoral was extracted separately to extract deep learning features by convolutional neural network

Since the biological basis of peritumoral radiomics features in the prognosis prediction of NSCLC is well established, we expanded the ROI segmentations into three peritumoral regions. After referencing existing studies, we finally selected peritumoral extension areas of 3 voxel units (ROI-3u), 6 voxel units (ROI-6u) and 12 voxel units (ROI-12u) (Fig. 3A). Peritumoral areas that expanded beyond the lung parenchyma were erased to prevent errors (Fig. 3B). After that, the maximum cross-section of ROI-tumoral was extracted separately to apply a convolutional neural network to extract deep learning features (Fig. 3C, D).

Pyradiomics in Python (version 3.7) was used to extract tumoral and peritumoral radiomics features from CT images, including first-order features, shape features (2D and 3D), gray level features (gray level cooccurrence matrix (GLCM), gray level size zone matrix (GLSZM), gray level run length matrix (GLRLM), neighbouring grey tone difference matrix (NGTDM) and gray level dependence matrix (GLDM) and wavelet features. The extracted features were normalized to a standard dataset with a mean of 0 and a variance of 1. Additionally, two pretrained ResNet18 models were used to extract 2D and 3D deep learning features from ROI-tumoral and to reduce the extracted features down to 50.

### Assessment of tumour spread through air spaces

Based on the 2015 World Health Organization classification of lung cancer and the study by Kadota et al., we developed the following criteria to define STAS: single tumour cells or clusters of tumour cells present in the alveolar space at least one alveolar septum away from the margin of the main body of the tumour. The exclusion criteria, as reported by Kadota et al., were as follows: (i) scattered tumour drifts or clusters of cells with rough margins due to cutting of the specimen, and (ii) clusters of tumour cells detached from the alveolar wall or interstitial lung parenchyma due to poor preservation.

**Fig. 4:**
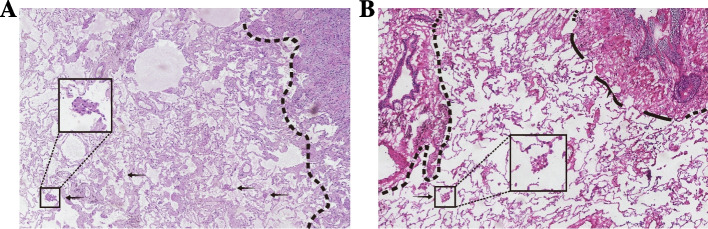
Microscopic view of spread through air spaces. **A**-**B** Assessment of tumor spread through air spaces by haematoxylin and eosin staining. Tumor cell masses were spread through the air spaces and located in the alveolar cavity beyond the margins of the main body of the tumor

Postoperative haematoxylin and eosin (HE)-stained sections of surgical specimens from all enrolled patients were reviewed for the presence of STAS by pathologists X.M.L (with 27 years of experience in pathology) and D.Y, who were blinded to the prognosis of the patients (Fig. 4). Ultimately, 118 patients (52.2%) in the development cohort and 25 patients (49.0%) in the validation cohort were determined to be STAS positive.

### Clinical outcome

The prognostic information of all enrolled patients was obtained through the electronic medical record system as well as by telephone follow-up. In this study, the endpoints were recurrence-free survival (RFS) and tumour recurrence. Tumour recurrence was confirmed by imaging or lymph node pathology through aspiration biopsy and by bronchoscopy for peripheral recurrence or distant metastasis recurrence. RFS was defined as the time between the date of surgery and the date on which tumour recurrence occurred or the date of the last follow-up visit if no recurrence occurred. For patients in the development cohort, the last follow-up date was September 1, 2021, and for patients in the validation cohort, the last follow-up date was December 1, 2022.

### Model construction and validation

For PyRadiomics-extracted features from ROI-tumoral, ROI-3u, ROI-6u and ROI-12u, we constructed a feature selection pipeline. First, a Spearman correlation test was performed for the extracted features and if paired features had a correlation greater than 0.9, then one of the features was randomly excluded. Then, least absolute shrinkage and selection operator (LASSO) regression was performed for the remaining features without strong correlations to filter out the prognosis-related features. Finally, a prognosis-related signature was constructed using multivariable Cox regression. For the 2D and 3D deep learning features extracted from the ResNet18 model, since the number of reduced features was already sufficiently small, we did not go through the first step of screening and directly performed LASSO regression to screen for prognosis-relevant features and constructed multivariable Cox regression signature. Prognostic risk scores were calculated using each of the six signatures, and these six risk scores were used with the presence of STAS as the final variable to construct the final multivariable Cox regression model: RAISm.

Finally, the models were evaluated in the training set, test set and validation set using receiver operating characteristics (ROC) curves, decision curve analysis (DCA) curves and Kaplan‒Meier (KM) curves, respectively, and the specificities and sensitivities of the models were calculated.

### Statistical analysis

Continuous variables of the baseline data following a normal distribution are expressed as means ± standard deviations. Continuous variables for which baseline data did not follow a normal distribution were presented as median values (Interquartile range).The optimal parameter configuration for the LASSO regression was determined by 50 cross-validations, retaining the features at lambda equal to the minimum value, except for the features of ROI-12u. Since the number of features retained at the minimum lambda was too many for ROI-12u, with more than 25 remaining after LASSO filtering, the features retained when lambda equals min + 1 se were retained. The DeLong test was used to determine the variability between multiple models. The high- and low-risk groups were determined based on the optimal cut-off values of the final model determined by the ROC curves. Survival curves were plotted using the Kaplan‒Meier method and compared between groups by the log-rank test. *P* values less than 0.05 were considered statistically significant. All statistical analyses were performed using R software (version 4.2.1).

## Results

### Patient baseline characteristics and STAS assessment results

To maintain the simplicity of the model, we did not include clinical information as variables in our model. The baseline characteristics of 226 patients in the development cohort and 51 patients in the validation cohort are presented in Table 1. We compared the population distribution of the development cohort of the model with the validation cohort except for the outcome metrics (RFS and RFS time). The results indicated no significant differences in information between the two cohorts, except for the gender distribution (*p* = 0.017). Since gender was not included in the study, it did not have an impact on the results. In the development cohort, 61.9% of patients were pathologically staged as IA and 38.1% as IB; according to the GRADE system, 54.0% of patients were classified as GRADE 1, 10.6% as GRADE 2 and 35.4% as GRADE 3; a total of 118 (52.2%) patients were evaluated as STAS positive; and the overall recurrence rate was 14.6%. In the validation cohort, 70.6% of patients had stage IA pathology, and 29.4% of patients had stage IB pathology; 60.8% of patients were classified as GRADE 1, 19.6% as GRADE 2 and 19.6% as GRADE 3; a total of 25 (49.0%) patients were evaluated as STAS positive; and the overall recurrence rate was 19.6%. The details of the clinical information are shown in Supplementary Table 1.

**Table 1:** Characteristics baseline of patients in the total cohort

	**Development Cohort (226)**		**Validation cohort (51)**	***P* value**
	**Training Set (164)**	**Test Set (62)**		
**Age**				0.543
**Mean ± SD**	61.1 ± 8.7	63.9 ± 6.4	60.7 ± 9.2	
**Gender**				**0.017**
**Male (%)**	80(48.8)	35(56.5)	36(70.6)	
**Female (%)**	84(51.2)	27(43.5)	15(29.4)	
**Pathological TNM stage**				0.319
**IA (%)**	102(62.2)	38(43.5)	36(70.6)	
**IB (%)**	62(37.8)	24(56.5)	15(29.4)	
**Grade stage**				0.062
**1(%)**	87(53.1)	35(56.5)	31(60.8)	
**2(%)**	15(9.1)	9(14.5)	10(19.6)	
**3(%)**	62(37.8)	18(29.0)	10(19.6)	
**STAS**				0.099
**Yes (%)**	91(55.5)	27(43.5)	26(51.0)	
**No (%)**	73(44.5)	35(56.5)	25(49.0)	
**RFS status**				-
**Relapse (%)**	25(15.2)	8(12.9)	10(19.6)	
**No relapse (%)**	139(84.8)	54(87.1)	41(80.4)	
**RFS time (Month)**				-
**Median (IQR)**	40.2(34.5–51.9)	40.9(35.5–50.5)	38.7(35.4–40.6)	

*SD* Standard deviation, *STAS* Spread through air spaces, *RFS* Recurrence free survival, *IQR* Interquartile range

### Feature extraction and selection

For each ROI, a total of 1133 features were extracted by PyRadiomics. After feature exclusion through the Spearman correlation test, 258 features from ROI-tumoral, 232 features from ROI-3u, 237 features from ROI-6u, and 237 features from ROI-12u were retained. For ROI-tumoral, 50 reduced deep learning 2D features and 50 reduced deep learning 3D features were extracted by the ResNet18 convolutional neural network. Afterwards, six prognosis-related signatures were constructed by screening with LASSO regression and multivariable COX regression (Rad-tumoral signature: 8 features from ROI-tumoral, Rad-peritumoral-3u signature: 2 features from ROI-3u, Rad-peritumoral-6u signature: 5 features from ROI-6u, Rad-peritumoral-12u signature: 11 features from ROI-12u, DeepL-2d signature: 12 2D deep learning features from ROI-tumoral, and DeepL-3d signature: 8 3D deep learning features from ROI-tumoral) (Supplementary Fig. 1). The details of the extracted features are shown in Supplementary Tables 2–7.

### Model development and validation

**Fig. 5:**
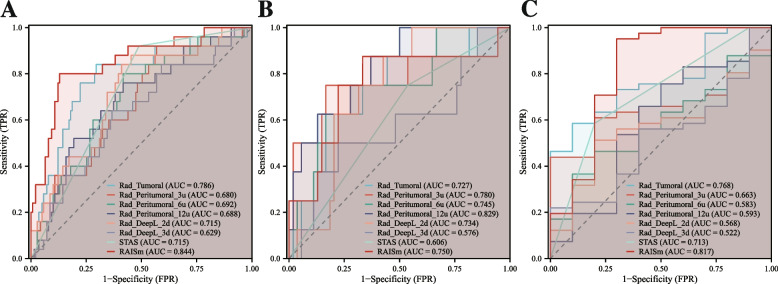
The performance of RAISm and single modality signatures in all cohorts. The ROC curves of RAISm and single modality signatures in **A** training cohort, **B** test cohort and **C** validation cohort

The prognostic risk scores obtained from each signature were calculated separately (Supplementary table 8), and these risk scores were used as variables along with the presence of STAS to construct the RAISm. After that, the performance of RAISm and the signatures were evaluated in each of the three cohorts (Fig. 5 and Table 2).

**Table 2:** Performance evaluation of the models in the development cohort and validation cohort

	**AUC (95%CI)**	**Sensitivity**	**Specificity**	**PPV**	**NPV**	**Accuracy**	**Youden Index**
**Training cohort**							
Rad-tumoral	0.785 (0.681–0.890)	0.840	0.705	0.339	0.961	0.726	0.545
Rad-peritumoral-3u	0.680 (0.568–0.792)	0.920	0.432	0.225	0.968	0.506	0.352
Rad-peritumoral-6u	0.685 (0.574–0.796)	0.800	0.583	0.256	0.942	0.616	0.383
Rad-peritumoral-12u	0.688 (0.576–0.800)	0.760	0.583	0.247	0.931	0.610	0.343
Rad-DeepL-2d	0.692 (0.579–0.805)	0.840	0.590	0.269	0.953	0.628	0.430
Rad-DeepL-3d	0.629 (0.509–0.750)	0.640	0.633	0.239	0.907	0.634	0.273
STAS	0.727 (0.654–0.799)	0.920	0.511	0.253	0.973	0.573	0.431
RAISm	0.847 (0.762–0.932)	0.800	0.871	0.526	0.960	0.860	0.671
**Test cohort**							
Rad-tumoral	0.727(0.533–0.921)	0.875	0.556	0.226	0.968	0.597	0.431
Rad-peritumoral-3u	0.780(0.560–1.000)	0.750	0.833	0.400	0.957	0.823	0.583
Rad-peritumoral-6u	0.745(0.552–0.939)	0.750	0.722	0.286	0.951	0.726	0.472
Rad-peritumoral-12u	0.829(0.685–0.972)	0.875	0.630	0.259	0.971	0.661	0.505
Rad-DeepL-2d	0.734(0.590–0.878)	0.875	0.574	0.233	0.969	0.613	0.449
Rad-DeepL-3d	0.576(0.333–0.819)	0.500	0.778	0.250	0.913	0.742	0.278
STAS	0.606(0.433–0.780)	0.750	0.463	0.171	0.926	0.500	0.213
RAISm	0.750(0.531–0.969)	0.875	0.667	0.280	0.973	0.694	0.542
**Validation cohort**							
Rad-tumoral	0.768(0.625–0.912)	0.585	0.900	0.960	0.346	0.647	0.485
Rad-peritumoral-3u	0.663(0.509–0.818)	0.439	1.000	1.000	0.303	0.549	0.439
Rad-peritumoral-6u	0.583(0.406–0.760)	0.366	0.900	0.938	0.257	0.471	0.266
Rad-peritumoral-12u	0.593(0.374–0.812)	0.659	0.600	0.871	0.300	0.647	0.259
Rad-DeepL-2d	0.568(0.390–0.746)	0.512	0.800	0.913	0.286	0.569	0.312
Rad-DeepL-3d	0.522(0.332–0.712)	0.220	1.000	1.000	0.238	0.373	0.220
STAS	0.713(0.555–0.872)	0.585	0.800	0.923	0.320	0.627	0.385
RAISm	0.817(0.625–1.000)	0.951	0.700	0.929	0.778	0.902	0.651

*AUC* Area under the receiver operating characteristics curve, *CI* Confidence interval, *PPV* Positive predictive values, *NPV* Negative predictive values, *Rad-tumoral* Tumoral radiomics signature, *Rad-peritumoral-3u* Peritumoral radiomic signature extracted from the 3 voxel units peritumoral area, *Rad-peritumoral-6u* Peritumoral radiomic signature extracted from the 6 voxel units peritumoral area, *Rad-peritumoral-12u* Peritumoral radiomic signature extracted from the 12 voxel units peritumoral area, *Rad-DeepL-2d* Radiomics signature constructed by 2d deep learning features, *Rad-DeepL-3d* Radiomics signature constructed by 3d deep learning features, *STAS* Spread through air spaces, *RAISm* RAdiomcs Integrated with STAS status model

The AUC of RAISm was 0.847 (95% confidence interval (CI), 0.762–0.932) in the training set, 0.750 (95% CI, 0.531–0.969) in the test set, and 0.817 (0.625–1.000) in the validation set. The Youden index of RAISm was 0.671 in the training set, 0.542 in the test set, and 0.651 in the validation set. Among all models, RAISm had the best performance in both the training and validation sets. In the test set, the model with the highest AUC was Rad-peritumoral-12u, with 0.829 (95% CI, 0.685–0.972), and the model with the highest Youden index was Rad-peritumoral-3u, with a value of 0.583. This might be because fewer patients in the test set relapsed (8, 12.9%), resulting in a vulnerable performance of the model.

Regarding DeLong’s test, RAISm had significantly different results from all other models, except for Rad-tumoral (Supplementary table 9) (*p* = 0.074); however, the performance of RAISm was still much better than that of the Rad-tumoral signature in terms of AUC, accuracy and Youden index.

### RAISm evaluation

**Fig. 6:**
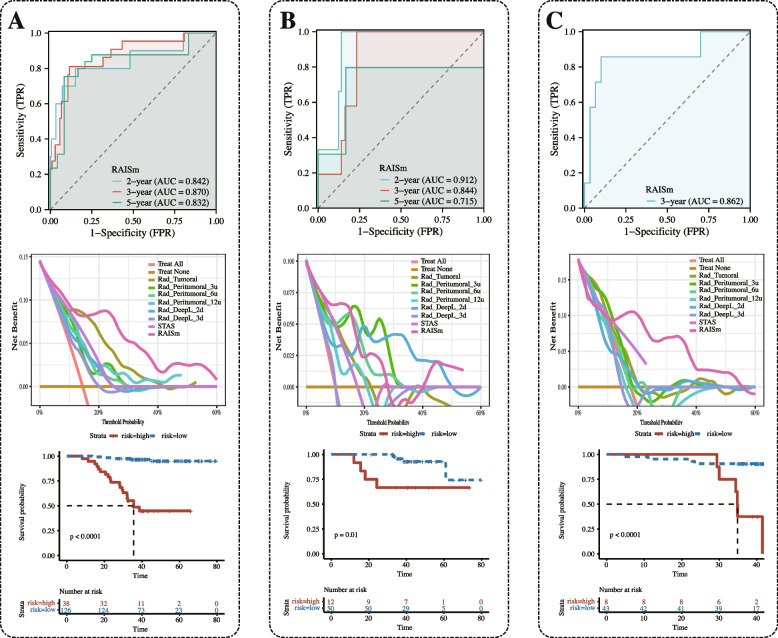
The performance of RAISm was evaluated in all cohorts. Time-dependent ROC, DCA curves and survival curves for RAISm high and low risk groups in **A** training cohort, **B** test cohort and **C** validation cohort

Finally, we visualized RAISm in the form of a nomogram (Supplementary Fig. 2) and evaluated it in the training set (Fig. 6A), test set (Fig. 6B) and validation set (Fig. 6C). We first plotted the time-dependent ROC curves of RAISm in the three cohorts. In the validation cohort, there was only one patient with > 3 years of follow-up and only two patients with < 2 years of follow-up, so we only plotted the ROC curve for 3 years. According to the time-dependent ROC curve, RAISm performed well, especially for predicting the 3-year recurrence rate, and showed high prediction accuracy (AUC = 0.870 in the training set, AUC = 0.844 in the test set, AUC = 0.862 in the validation set). DCA curves were also used to evaluate the applicability of the model for clinical decision making. The results showed that RAISm had the best clinical net benefit in both the training and validation sets. The best-performing model in the test set was Rad-DeepL-2d. Then, based on the best cut-off value (2.390) from the model determined in the training set, the patients in the training set, test set and validation set were all divided into a high-risk group and a low-risk group, and RFS survival curves were plotted for the two groups. The results showed that RAISm had an excellent performance in stratifying recurrence risk in all three cohorts (*p* < 0.001 in the training set, *p* = 0,010 in the test set, *p* < 0.001 in the validation set).

**Fig. 7:**
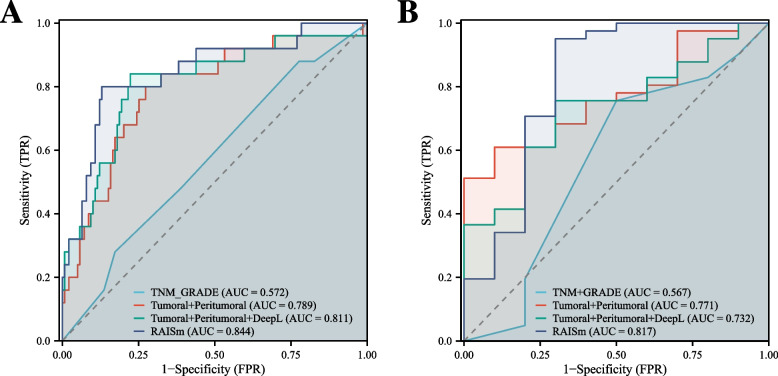
Comparison of Model performance of RAISm and combinations of signatures. The ROC curve of RAISm, handcrafted radiomics model, all-radiomics model and TNM + GRADE model in **A** training cohort, **B** test cohort and **C** validation cohort

We also compared RAISm with the most commonly used current clinical markers for assessing LUAD prognosis, namely, TNM staging and the GRADE system. The results show that RAISm could identify prognostic risk far better than the TNM staging guidelines and the GRADE system (Fig. 7). In addition to comparing the performances of RAISm and individual signatures, we also compared performance between RAISm and combinations of signatures in the training set (Fig. 7A) and validation set (Fig. 7B). The results showed that RAISm still had the best discrimination relative to the model incorporating only handcrafted radiomics features and the model incorporating all radiomics features. This meant that omitting any of the feature sets would have some impact on the final model performance.

## Discussion

In the present study, we developed a prediction model for the early recurrence of stage I LUAD based on a combination of machine learning-based radiomics features from preoperative CT and the presence of STAS. The final combined model RAISm was accurate in predicting the 2-year and 3-year recurrence rates of stage I LUAD, with a favourable AUC and high sensitivity, specificity, NPV and PPV in both the development cohort and external validation cohort, and had a superior performance to conventional single-modality models. Our study provides a reproducible and reliable tool for prognostic assessments that facilitates adjustments to treatment strategies for patients with early-stage LUAD and enables the clinical implementation of computer-assisted personalized management of patients with early-stage LUAD.

To maximize lung function and minimize complications, the surgical treatment strategy for early-stage lung adenocarcinoma is still based on sublobar resection [13]. However, several studies have noted that patients with STAS-positive stage I LUAD who underwent sublobar resection had a significantly higher postoperative recurrence rate than those who underwent lobectomy [14]. Therefore, lobectomy is currently recommended for patients with STAS-positive stage I LUAD. However, how to more accurately identify patients at high risk for early recurrence is important for individualized patient management. Our study found that combining radiomics features with the presence of STAS can more accurately identify patients at high risk of recurrence who may be suitable for a more aggressive postoperative treatment strategy.

Tumoral radiomics features have been widely used for the prognostic prediction of lung adenocarcinoma [15]. However, few studies have applied peritumoral imaging features to assist in such predictions, and the choice of peritumoral region remains controversial. Recently, Wu et al. confirmed that peritumoral radiomics features based on CT images are reliable for predicting the prognosis of NSCLC [16]. This study also noted that the peritumoral region was best defined as extensions from the tumour boundary of 15 mm, 20 mm, or 30 mm. However, there are no studies that give advice on the range of peritumoral areas that is best for predicting prognosis. Chen et al. [17] constructed models by extracting radiomics features from regions measuring 3 mm, 6 mm, and 9 mm from the tumour margin and showed that the prognostic signatures constructed based on the radiomics features extracted from the 9 mm region around the tumour had the highest AUC in the training (0.82) and validation (0.67) sets. Another study by Lin et al. [18] also extracted radiomics features from the 3-mm and 6-mm peritumoral regions and showed that the features from the 3-mm region had a higher predictive accuracy. In a study conducted by Chang et al. [19] using radiomics to predict chemotherapy response, the features from the 3–6 mm peritumoral region had the highest predictive accuracy among those extract from the 3–6 mm, 6–9 mm and 9–12 mm peritumoral regions. It can be seen that investigators have chosen different peritumoral ranges, but the best-performing peritumoral signatures are basically composed of features in the 3–9 mm peritumoral range. Therefore, based on this evidence, we selected 3 voxel units (2.1 mm), 6 voxel units (4.2 mm), and 12 voxel units (8.4 mm) as the peritumoral regions. However, according to the results of DeLong’s test in training cohorts, the accuracies of the prognostic prediction signatures constructed based on the radiomics features from these three regions were not significantly different (Supplementary table 9). In fact, in addition to this we extracted the radiomics features of 9 voxel units in the perineurium and processed them with the same feature screening process. However, no features were retained at both lambda = min and lambda = min + 1se during LASSO regression. This suggests that the radiomics features of peritumoral-9u may be poorly used for prognostic prediction.

Tunali et al. [20] found that the stability and reproducibility of wavelet features extracted from the peritumoral region were poor in survival models constructed based on radiomics features. The best-performing features in survival models tend to be those that were stable and reproducible, and these features can enhance the reproducibility of the study and reduce overfitting. This explains why among all of the models we constructed, Rad-peritumoral-3u, Rad-peritumoral-6u and Rad-peritumoral-12u were less effective: Rad-peritumoral-3u was composed of 2 wavelet features, while Rad-peritumoral-6u had 4 wavelet features out of its 5 predictors, and Rad-peritumoral-12u had 7 wavelet features out of its 11 predictors. In addition, the study [20] noted that whether the peritumoral ROI region strictly covered the lung parenchyma (e.g., the ROI region went beyond the lung parenchyma and covered the heart) had no effect on the stability of the features. However, in pursuit of logical interpretability and optimal performance of the model, we still chose to retain only the ROI covering the parenchymal portion of the lung, although we retained the part of pleural indentation.

ResNet18, a classical convolutional neural network (CNN), has been widely used in medical image recognition and semantic segmentation. ResNet, as a deep residual network architecture, is characterized by the introduction of "jump connections", i.e., adding a cross-layer connection in each residual module, so that the information can be directly passed to the later convolutional layers, preserving the original features and avoiding the disappearance of features layer by layer. Therefore, it has advantages as a feature extraction tool that other CNNs do not have. Several radiomics-related studies have already used ResNet for feature extraction, which indicates that its application in medical image feature extraction is relatively mature [21–25]. We used a ResNet18 model pretrained by ImageNet [26], a large computer vision dataset, to extract deep learning features. From the results, it can be seen that the deep learning features do not have a significant advantage in terms of prediction accuracy over the conventional radiomics features extracted by PyRadiomics (Supplementary table 9). Even in the validation set, the model incorporating only conventional radiomics features outperformed the model incorporating deep learning features. Not coincidentally, in the study conducted by Feng and colleagues [11], the models constructed using deep learning features extracted from VGG19 had the lowest AUC compared to models constructed by other nonmachine learning methods. In a multicentre cohort study designed by Cui et al. [27], features extracted by a deep learning model combined with handcrafted radiomics features were used to construct a nomogram for predicting the efficacy of neoadjuvant chemotherapy in advanced gastric cancer. Although the AUC of the deep learning model was better than that of the handcrafted model in the training set, the model constructed from the handcrafted model outperformed the deep learning model and had a higher AUC in the two external validation cohorts. However, in the above studies, the models based on a combination of deep learning models and other models all performed better than the individual models alone, and this was also true in our study. The models constructed with deep learning features were not superior to the models constructed with conventional handcrafted radiomics features, but the models that were combined with deep learning features had a better performance.

STAS has received much attention since it was proposed. It has been found that STAS in sublobar resection is closely associated with locoregional recurrence of lung cancer [28]. Therefore, lobectomy is now recommended for lung cancer patients with STAS [14, 29]. Nevertheless, it has also been found that STAS is an independent prognostic factor influencing both limited and radical resection [30]. However, preoperative evaluations of STAS are difficult, and it is controversial whether intraoperative resection contributes to the development of STAS, so most STAS is only detected postoperatively. Nevertheless, we believe that further risk stratification according to whether patients were postoperatively determined to have STAS is important and relevant for further patient treatment decisions. From our study, STAS had the second highest performance among all single-modality models, after Rad-tumoral signatures, in both the training and validation sets. In fact, during the construction of RAISm, we found that only the Rad-tumoral risk score and the presence of STAS were independent risk factors in the multivariable Cox regression. In the validation set, the AUC of RAISm was improved by 0.085 over that of the model incorporating only radiomics features. This suggested that the inclusion of STAS was of indispensable importance to the performance of the final RAISm model. However, despite the clear definition of STAS, the assessment of STAS is still not an easy task. In actual evaluations of STAS, the results can be confounded by many factors, such as poor quality of the sections, improper preservation of the sections, and the presence of macrophage clumps in the alveoli. Therefore, our assessment may be subject to some errors, and more studies may be required to further standardize and unify the assessment methods of STAS.

We reviewed the currently published radiomics models for prognostic prediction constructed using real-world data for early LUAD [31–35]. We found that these models were basically constructed using either tumoral radiomics features alone or peritumoral radiomics features alone, combined with some clinical information and morphological features. The sample sizes of these studies ranged from 119 to 295. The largest sample size was in a study by Zhang et al. [31], which used a radiomics model to assess the prognostic risk of patients with postoperative lung adenocarcinoma, and the C-index of the multivariable Cox regression model constructed using a radiomics risk score combined with clinical features was 0.71 in a training set that included 217 patients with postoperative LUAD. The study also compared a deep learning model with a handcrafted radiomics model and found that the CNN-based deep learning model was not as effective as the handcrafted radiomics model in predicting prognosis. Notably, in a single-centre retrospective study by Kirienko et al. [35], the investigators constructed a machine learning-based radiogenomic model using radiomics features extracted from [18F] FDG PET/CT in combination with the gene expression profile, and the model showed an excellent prediction ability (AUC = 0.87). However, the radiogenomic data used in the study were from only 74 samples and were not validated in other datasets. Moreover, the cost-effectiveness of the model was significantly reduced after the inclusion of gene sequencing. Compared to the above studies, our study constructed a superior radiomics-pathology model with a more adequate sample size (*n* = 277) using the most commonly used postoperative clinical and surgical thoracic examinations.

The design of our study was informed by the TRIPOD statement, which is a guideline for multivariable prediction models for individual prognosis [36]. In addition, we quality controlled and scored our study using the radiomics quality scoring (RQS) system [37]. According to a systematic review of the efficacy of radiomics prediction models for non-small cell lung cancer (NSCLC) conducted by Chetan et al. [38], the median RQS score of the currently published radiomics models for NSCLC is +2.5 (range -5 to 9). In contrast, the RQS score for RAISm resulted was +16 (Supplementary Fig. 3). The main limitation was the lack of prospective validation. This indicated the high quality of our study relative to the currently published radiomics studies in NSCLC.

However, our study still has some limitations. First, this study was a retrospective study due to the long follow-up period. Second, the performance of RAISm in the test set reflects that our sample size might need further expansion. Only 8 of the 62 individuals in the test set reached the study endpoint during the follow-up period, which resulted in a very small number of prediction errors for the model, which could seriously affect its performance. However, the model performed well in the external validation set, thus partially mitigating this limitation. In addition, the demographics and ethnicity of the samples are limited, and future validation using multi-ethnic samples will be required. Third, although radiomics features extracted through manual segmentation have high prediction accuracy, the process has high time and labour costs. However, with the development and maturity of automatic medical image segmentation technology, this problem will eventually be addressed. In the future, our work will mainly focus on further refining and validating RAISm in high-quality, multicentre, prospective studies.

## Conclusion

In conclusion, in this retrospective cohort study, we pioneered the combination of preoperative CT-based radiomics with the presence of STAS determined by postoperative pathology to develop a model for predicting postoperative metastasis of stage I lung adenocarcinoma and confirmed the superior predictive effect of the model in both internal and external validation sets, showing that the model can assist in the development of postoperative treatment strategies for patients with stage I lung adenocarcinoma.

### Supplementary Information

**Additional file 1: Supplementary figure 1.** The final features in the six signature models selected by LASSO-COX regression. **Supplementary figure 2.** The nomogram obtained by visualizing RAISm. **Supplementary figure 3.** The RQS score of RAISm.

**Additional file 2: Supplementary table 1.** The STAS status and clinical outcome of enrolled patients. **Supplementary table 2.** Radiomic features extracted from ROI-tumoral by Pyradiomics. **Supplementary table 3.** Radiomic features extracted from ROI-3u by Pyradiomics. **Supplementary table 4.** Radiomic features extracted from ROI-6u by Pyradiomics. **Supplementary table 5.** Radiomic features extracted from ROI-12u by Pyradiomics. **Supplementary table 6.** 2D Deep learning radiomics features from ROI-tumoral by Convolutional Neural Networks. **Supplementary table 7.** 3D Deep learning radiomics features from ROI-tumoral by Convolutional Neural Networks. **Supplementary table 8.** The final features in the six signature models selected by LASSO-COX regression and the weight of them. **Supplementary table 9.** The results of DeLong’s test in different models.

## Data Availability

All numerical data generated or analysed during this study are included in this published article [and its supplementary information files]. The imaging data used and/or analysed during the current study are available from the corresponding author on reasonable request.

## Abbreviations

CT: Computerized tomography
LUAD: Lung adenocarcinoma
STAS: Spread through air spaces
RAISm: RAdiomics Integrated with STAS status model
DICOM: Digital Imaging and Communications in Medicine
PACS: Picture Archiving and Communication System
ROI: Region of interest
GLCM: Gray Level Co-occurrence Matrix
GLSZM: Gray Level Size Zone Matrix
GLRLM: Gray Level Run Length Matrix
NGTDM: Neighbouring Gray Tone Difference Matrix
GLDM: Gray Level Dependence Matrix
RFS: Recurrence-free-survival
LASSO: Least absolute shrinkage and selection operator
ROC: The receiver operating characteristics curve
AUC: Area under curve
DCA: Decision curve analysis
CI: Interval of confidence
NPV: Negative predictive values
PPV: Positive predictive values
CNN: Convolutional neural network
SD: Standard deviation
IQR: Interquartile range
