# Supplementary material for: Preoperative CT-based radiomics combined with tumour spread through air spaces can accurately predict early recurrence of stage I lung adenocarcinoma: a multicentre retrospective cohort study
Source: Cancer Imaging. 2023 Sep 7;23:83. doi: 10.1186/s40644-023-00605-3 (PMC10485937; doi:10.1186/s40644-023-00605-3)
Supplement: Supplementary file 1 — Additional file 1: Supplementary figure 1. The final features in the six signature models selected by LASSO-COX regression. Supplementary figure 2. The nomogram obtained by visualizing RAISm. Supplementary figure 3. The RQS score of RAISm. [file 40644_2023_605_MOESM1_ESM.pdf]

Supplementary Figure 1

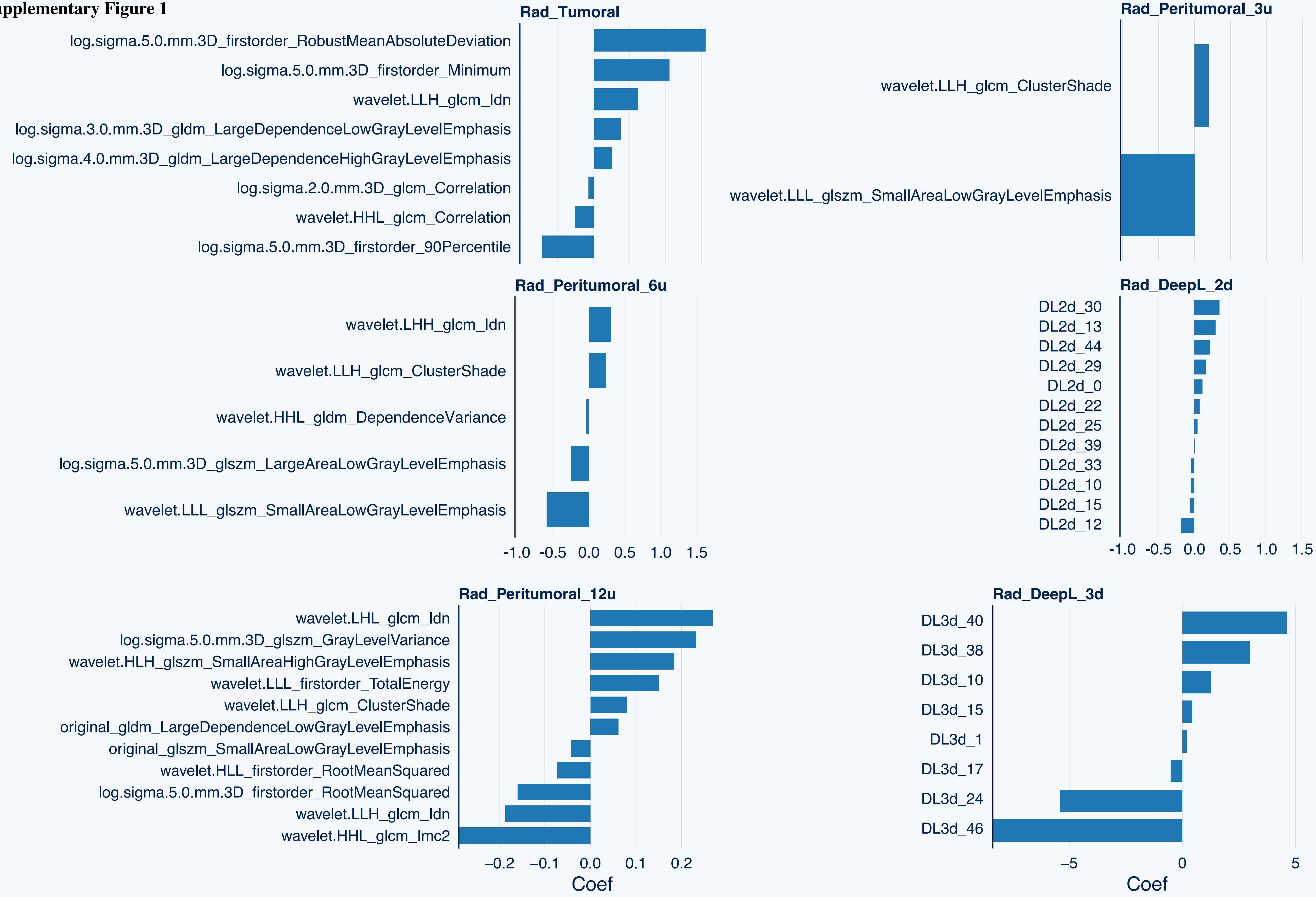

Supplementary Figure 2

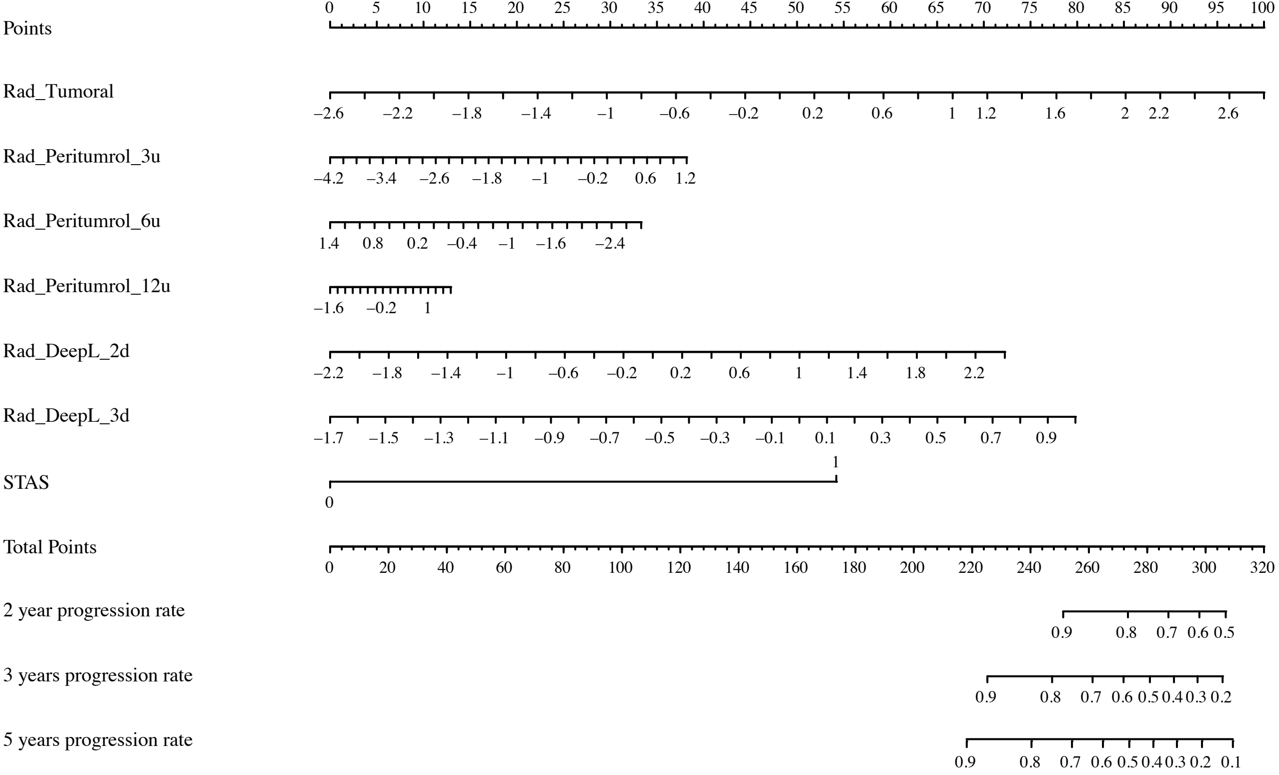

Supplementary Figure 3

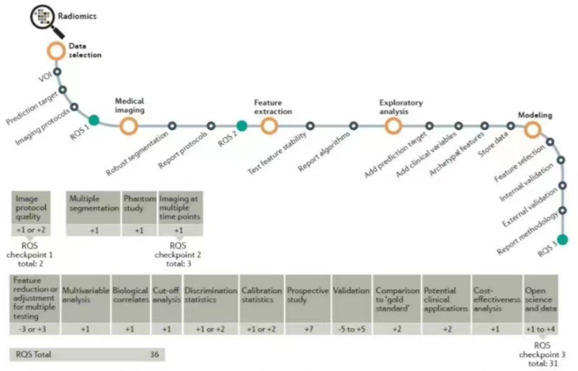

Figure 1 | **Flowchart depicting the workflow of radiomics and the application of the RQS.** The workflow includes the necessary steps in a radiomic analysis. The RQS both rewards and penalizes the methodology and analyses of a study, consequently encouraging the best scientific practice. RSQ, radiomics quality score; VOI, volume of interest.

Image protocol quality - well-documented image protocols (for example, contrast, slice thickness, energy, etc.) and/or usage of public image protocols allow reproducibility/replicability	☑ protocols well documented<br>☐ public protocol used<br>☐ none
Multiple segmentations - possible actions are: segmentation by different physicians/algorithms/software, perturbing segmentations by (random) noise, segmentation at different breathing cycles. Analyse feature robustness to segmentation variabilities	◉ yes<br>○ no
Phantom study on all scanners - detect inter-scanner differences and vendor-dependent features. Analyse feature robustness to these sources of variability	◉ yes<br>○ no
Imaging at multiple time points - collect images of individuals at additional time points. Analyse feature robustness to temporal variabilities (for example, organ movement, organ expansion/shrinkage)	○ yes<br>◉ no
Feature reduction or adjustment for multiple testing - decreases the risk of overfitting. Overfitting is inevitable if the number of features exceeds the number of samples. Consider feature robustness when selecting features	◉ Either measure is implemented<br>○ Neither measure is implemented
Multivariable analysis with non radiomics features (for example, EGFR mutation) - is expected to provide a more holistic model. Permits correlating/inferencing between radiomics and non radiomics features	◉ yes<br>○ no
Detect and discuss biological correlates - demonstration of phenotypic differences (possibly associated with underlying gene–protein expression patterns) deepens understanding of radiomics and biology	○ yes<br>◉ no
Cut-off analyses - determine risk groups by either the median, a previously published cut-off or report a continuous risk variable. Reduces the risk of reporting overly optimistic results	◉ yes<br>○ no
Discrimination statistics - report discrimination statistics (for example, C-statistic, ROC curve, AUC) and their statistical significance (for example, p-values, confidence intervals). One can also apply resampling method (for example, bootstrapping, cross-validation)	☑ a discrimination statistic and its statistical significance are reported<br>☐ a resampling method technique is also applied<br>☐ none
Calibration statistics - report calibration statistics (for example, Calibration-in-the-large/slope, calibration plots) and their statistical significance (for example, P-values, confidence intervals). One can also apply resampling method (for example, bootstrapping, cross-validation)	☐ a calibration statistic and its statistical significance are reported<br>☐ a resampling method technique is applied<br>☑ none
Prospective study registered in a trial database - provides the highest level of evidence supporting the clinical validity and usefulness of the radiomics biomarker	○ yes<br>◉ no
Validation - the validation is performed without retraining and without adaptation of the cut-off value, provides crucial information with regard to credible clinical performance	☐ No validation<br>☑ validation is based on a dataset from the same institute<br>☐ validation is based on a dataset from another institute<br>☐ validation is based on two datasets from two distinct institutes<br>☐ the study validates a previously published signature<br>☐ validation is based on three or more datasets from distinct institutes
Comparison to 'gold standard' - assess the extent to which the model agrees with/is superior to the current 'gold standard' method (for example, TNM-staging for survival prediction). This comparison shows the added value of radiomics	○ yes<br>◉ no
Potential clinical utility - report on the current and potential application of the model in a clinical setting (for example, decision curve analysis).	◉ yes<br>○ no
Cost-effectiveness analysis - report on the cost-effectiveness of the clinical application (for example, QALYs generated)	○ yes<br>◉ no
Open science and data - make code and data publicly available. Open science facilitates knowledge transfer and reproducibility of the study	☑ scans are open source<br>☑ region of interest segmentations are open source<br>☐ the code is open sourced<br>☑ radiomics features are calculated on a set of representative ROIs and the calculated features and representative ROIs are open source

Total score **16** (44.44%)
